# Safety, tolerability, pharmacokinetics, and pharmacodynamics of a soluble guanylate cyclase stimulator, HEC95468, in healthy volunteers: a randomized, double-blinded, placebo-controlled phase 1 trial

**DOI:** 10.3389/fphar.2024.1359939

**Published:** 2024-06-12

**Authors:** Yu-zhou Gui, Wei Wang, Qing-qing Wu, Qi-chen Ding, Hong-jie Qian, Qiu-bei Lu, Ying-jun Zhang, Yu-lei Zhuang, Li Deng, Ying-lin Zuo, Lin Luo, Jing-ying Jia

**Affiliations:** ^1^ Shanghai Xuhui Central Hospital / Xuhui Hospital, Fudan University, Shanghai, China; ^2^ Shanghai Engineering Research Center of Phase I Clinical Research & Quality Consistency Evaluation for Drugs, Shanghai, China; ^3^ HEC R&D Center, Sunshine Lake Pharma Co, Ltd, Dongguan, China

**Keywords:** HEC95468, soluble guanylate cyclase stimulator, safety, pharmacokinetics, pharmacodynamics, healthy volunteers

## Abstract

Heart failure is the most costly cardiovascular disorder. New treatments are urgently needed. This study aims to evaluate the safety, pharmacokinetics, and pharmacodynamic profile of HEC95468, a soluble guanylate cyclase (sGC) stimulator, in healthy volunteers. Sixty-two, eighteen, and forty-eight participants were enrolled in the single ascending dose (SAD) study, the food effect (FE) study, and the multiple ascending dose (MAD) study, respectively. The study conforms to good clinical practice and the Declaration of Helsinki. Overall, HEC95468 was safe and tolerable; a higher proportion of HEC95468-treated participants reported mild headaches, dizziness, decreased blood pressure, increased heart rate, and gastrointestinal-related treatment-emergent adverse events (TEAEs), similar to the sGC stimulators riociguat and vericiguat. In terms of pharmacokinetic parameters, the maximum observed plasma concentration (C_max_) and the area under the concentration-time curve (AUC_0-t_) were dose-proportional over the dose range. Moderate accumulation was observed after multiple administrations of HEC95468. Systolic blood pressure (SBP) and diastolic blood pressure decreased, while 3′,5′-cyclic guanosine monophosphate (cGMP) concentration in plasma increased and heart rate was induced. Vasoactive hormones (renin, angiotensin II, and norepinephrine) in plasma were compensatorily elevated after oral administration. These data supported further clinical trials of HEC95468 in the treatment of heart failure and pulmonary arterial hypertension.

**Systematic Review Registration:** http://www.chinadrugtrials.org.cn, identifier CTR20210064.

## Introduction

Heart failure (HF) is a complex clinical syndrome characterized by dyspnea, fatigue, and fluid retention (Varughese, 2007). The prevalence of heart failure in the age group of 35–74 years in China is 0.9% (Cai et al., 2023). With the acceleration of population aging and the increasing diagnosis rate of common cardiovascular diseases such as hypertension and coronary heart disease, the occurrence of heart failure is gradually increasing (Nieminen et al., 2005). Multiple mechanisms are involved in the development of heart failure, including activation of the neuroendocrine system, changes in the vascular system, inflammatory response, cardio-renal syndrome, oxidative stress, myocardial factors, myocardial damage, matrix, and histiocytic remodeling (Greene et al., 2023a). Although significant advances have been made in the treatment of drugs and devices for heart failure over the past 20 years (Inciardi et al., 2022; Olivotto et al., 2023; Packer and Butler, 2023; Packer et al., 2023), the re-hospitalization rate of patients with heart failure remains high. The 5-year survival rate of HF patients remains low (<20%) (Sachdev et al., 2023).

In HF patients, insufficient soluble guanylate cyclase (sGC) and a reduction of cyclic guanosine monophosphate (cGMP) synthesis may occur and result from endothelial dysfunction and the increase of reactive oxygen species (Lombardi et al., 2021). This abnormality was associated with coronary microvascular dysfunction and myocardial derangements in HF (Tsao et al., 2023). Current standard-of-care (SOC) treatment, including angiotensin-converting enzyme (ACE) inhibitors, angiotensin receptor blockers (ARBs), mineralocorticoid receptor antagonists, or beta-blockers, could not alleviate the cardiac dysfunction mediated by the NO–sGC–cGMP pathway (Greene et al., 2023b). Direct stimulators of the sGC are therefore a new approach to addressing cGMP deficiency and could benefit patients with HF (Butler et al., 2022).

HEC95468 is a novel oral sGC stimulator developed as a potential best-in-class therapy for the treatment of HF and pulmonary arterial hypertension (PAH). In the preclinical experiments, HEC95468 exhibited satisfactory safety and pharmacokinetic profiles in rats and beagle dogs and exerted definite pharmacological effects on the rat heart failure model and PAH model (data not shown). Therefore, HEC95468 has been approved for clinical trials by the National Medicinal Product Agency (NMPA). This study aimed to investigate the safety and tolerability of HEC95468 and assess the pharmacokinetic and pharmacodynamic profiles in healthy Chinese volunteers.

## Methods

### Study design

**FIGURE 1:**
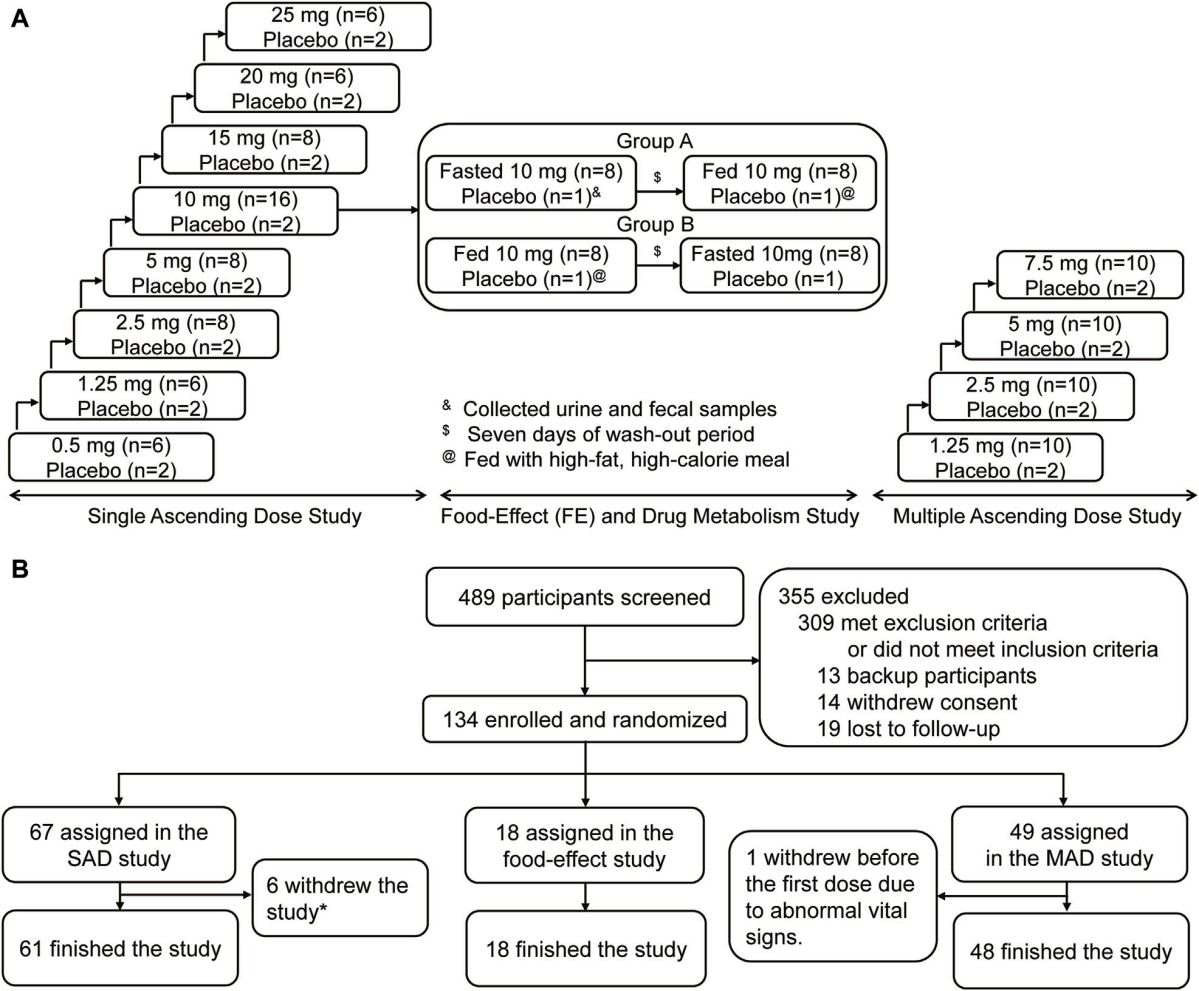
Study design and CONSORT diagram. **(A)** Study design. **(B)** CONSORT diagram.

This was a single-centered, randomized, double-blinded, placebo-controlled, single, and multiple ascending dose study aiming to evaluate the safety, tolerability, pharmacokinetics, and pharmacodynamics of HEC95468 tablets in healthy volunteers. The study consisted of three parts: a single ascending dose (SAD) study, a food effect (FE) and drug metabolism study, and a multiple ascending dose (MAD) study. The SAD study comprised eight dose levels: 0.5 mg, 1.25 mg, 2.5 mg, 5 mg, 10 mg, 15 mg, 20 mg, and 25 mg groups. The FE and drug metabolism were jointly evaluated in the 10 mg group. The MAD study evaluated four dose levels: 1.25 mg, 2.5 mg, 5 mg, and 7.5 mg groups. The study diagram is illustrated in Figure 1A.

### Ethics

The study was carried out by the Phase I Clinical Research Center of Shanghai Xuhui Central Hospital from March 2021 to January 2023. The study was registered at http://www.chinadrugtrials.org.cn (Registration No.: CTR20210064). The study protocol was approved by the Ethics Committee of the Shanghai Xuhui Central Hospital, and the study was conducted following the Declaration of Helsinki and Good Clinical Practice. All participants were required to provide a written informed consent form (ICF) before any study-related procedure was performed.

### Participants

Participants who met the following inclusion criteria but did not meet the exclusion criteria were eligible to participate in the study. The key inclusion criteria included healthy males or females between 18 and 45 years of age; body weight ≥50.0 kg for males and ≥45.0 kg for females; body mass index (BMI) in the range of 18.0–28.0 kg/m^2^; normal vital signs, physical examination, 12-lead ECG, and results of laboratory tests (including creatinine kinase) or abnormality with no clinical significance; no birth plan and voluntary effective contraceptive measures during the trial and within 3 months after administration; sitting blood pressure should be within 90 mmHg–140 mmHg for systolic blood pressure (SBP) and 60 mmHg – 90 mmHg for diastolic blood pressure (DBP); and the sitting heart rate should be within 50 bpm–90 bpm. The key exclusion criteria included a history of drug/excipient allergies; history of any systemic disorders or diseases; history of orthostatic hypotension; any concomitant medication within 2 weeks before dosing; participation in any clinical trial within 3 months; drug or alcohol addicts (more than 21 unit per week; 1 unit = 360 mL beer or 45 mL 40% liquor or 150 mL wine) or heavy smokers (more than 10 cigarettes per day); consumption of foods that may affect drug interactions (e.g., tea, coffee, and grapefruit) within 48 h before the first administration of HEC95468; history of blood donation/blood loss for more than 400 mL; positive tests for hepatitis B, hepatitis C, HIV, or syphilis; abnormality with clinical significance in chest X-ray; and positive pregnancy test for females.

### Dose level justification

HEC95468 tablets and placebo tablets (HEC R&D Center, Sunshine Lake Pharma Co, Ltd, Dongguan, Guangdong, China) had the same appearance. The dosage form of HEC95468 tablets was 0.25 mg, 1.25 mg, and 5 mg. The dose levels of HEC95468 were carefully selected according to the non-clinical pharmacology and toxicology data of HEC95468, as well as the clinical data of vericiguat and riociguat. In the rat model of heart failure and pulmonary hypertension, the effective dose was 1 mg/kg/day. The human equivalent dose was 9.6 mg/day. In the non-clinical pharmacology studies, the no observed adverse effect level (NOAEL) was 10 mg/kg in SD rats and 3 mg/kg in Beagle dogs. The human equivalent dose was 96 mg/day and 97.2 mg/day, respectively. The clinical dose of vericiguat for treating heart failure (heart failure with reduced ejection fraction and heart failure with preserved ejection fraction) is 10–15 mg/day (Yogasundaram et al., 2023). The clinically effective dose of riociguat in PAH indications is 3.0 mg/day–7.5 mg/day (Rosenkranz et al., 2015). Since the *in vitro* activity of HEC95468 was slightly higher than vericiguat and slightly lower than riociguat, the effective dose range of HEC95468 was estimated at 3–15 mg/day (QD). To guarantee clinical safety and a sufficient dose range for future clinical trials, 0.5 mg was set as the starting dose, while 25 mg was set as the highest level. Eight dose groups were designed in the SAD study: 0.5 mg, 1.25 mg, 2.5 mg, 5 mg, 10 mg, 15 mg, 20 mg, and 25 mg. The dose level of the food-effect study was set at 10 mg, which was the expected effective dose. The MAD study consisted of 4 dose regimens: 1.25 mg, 2.5 mg, 5 mg, and 7.5 mg for 9 consecutive days of administration.

## Objectives

The primary objective was to assess the safety and tolerability of HEC95468 in healthy Chinese participants. The secondary objective was to evaluate the pharmacokinetics and pharmacodynamics of HEC95468 in healthy Chinese volunteers. The exploratory objective was to assess the effect of food on the pharmacokinetics of HEC95468 and estimate the drug metabolism and transformation in healthy volunteers.

### Randomization and masking

After participants signed the ICF, the investigator assigned a screening number according to the arrival sequence at the site. The random numbers of the participants were generated by an independent statistician unrelated to this study. The independent statistician used SAS version 9.4 of the PLAN process, using the block random method to generate random numbers. Since this trial was a double-blind trial, the participants, investigators, and anyone involved in the analysis or interest of the trial were not aware of the allocation of the investigational drug.

### Procedures

For the SAD study, eligible participants were admitted to the Phase I unit 1 day before drug administration. On the morning of Day 1, after overnight fasting for >10 h, participants were administered HEC95468 tablets or placebo at the respective dose level with 240 mL of water under fasted conditions. All the participants stayed at the Phase I unit until 120 h post-dose. A follow-up visit by telephone was performed by investigators on Day 10 post-dose. In the FE study, eligible participants were randomized into two groups (Group A and Group B). In the first treatment period, Group A was administered 10 mg of HEC95468 after a >10h overnight fast, while Group B was administered 10 mg of HEC95468 after consumption of high-fat and high-calorie meals (total calories: approximately 800 kcal–1000 kcal, which derived approximately 150, 250, and 500–600 kcal from protein, carbohydrate, and fat, respectively). After a 7-day washout period, the second treatment period was conducted. Groups A and B reversed the treatment with each other. All the participants stayed at the site until 120 h post-dose. A follow-up visit by telephone was performed by investigators on Day 10 after the last dose. For the MAD study, eligible participants were admitted to the Phase I unit 1 day before drug administration. On the morning of dosing days, HEC95468 tablets or placebos at the respective dose level were administered within 30 min after overnight fasting for ≥10 h with 240 mL of water under fasted conditions. All the participants were dosed for nine consecutive days at once daily, and they stayed at the site until 120 h after the last dose. A follow-up visit by telephone was performed by investigators on Day 10 after the last dose.

### Safety and tolerability assessment

Safety was assessed by vital signs (blood pressure, heart rate, respiratory rate, and body temperature), physical examinations, clinical laboratory tests, 12-lead ECGs, and monitoring for adverse events (AEs) throughout the study. AEs were evaluated according to the National Cancer Institute Common Terminology Criteria for Adverse Events (CTCAE, version 5.0) and were managed and recorded promptly by qualified investigators according to relevant regulations. All AEs were coded using the Medical Dictionary for Regulatory Activities (MedDRA, version 25.1). The incidence of AEs was calculated by dividing the total number of participants who experienced at least one AE by the total number of participants. Treatment-emergent adverse events (TEAEs) were defined as AEs from the first administration of the investigational drug to the completion of central follow-up on day 10 ± 1 after the last dose. TEAEs related to investigational drugs were defined as related, likely to be related, and likely to be related to the causal relationship of investigational drugs. If a drug-related association could not be determined or was missing, the AE would be counted in drug-related TEAEs at the time of aggregation.

Dose escalation should be terminated when 1) ≥ 50% of participants experienced Grade 2 or above AEs; ≥ 33.3% of participants experienced Grade 3 or above AEs in a single-dose group; 2) under the premise of fully protecting the rights and interests of the participant and safety, the sponsor requests termination; and 3) the NMPA or the ethics committee orders the termination of the dose escalation.

### Pharmacokinetic assessment

Blood samples were collected at specific times before and after administration for pharmacokinetic assessment. For the SAD study, 3 mL of EDTA-K_2_ anticoagulated whole blood samples were collected at pre-dose and 0.25 h, 0.5 h, 0.75 h, 1 h, 1.5 h, 2 h, 3 h, 4 h, 6 h, 8 h, 10 h, 12 h, 24 h, 36 h, 48 h, 72 h, 96 h, and 120 h post-dose in the 0.5 mg, 1.25 mg, 2.5 mg, and 5 mg dose groups, which was the same as in the FE study. In addition, 3 mL of EDTA-K_2_ anticoagulated whole blood samples at 10 h and 36 h post-dose were omitted in the 15 mg, 20 mg, and 25 mg dose groups of the SAD study after the approval of the protocol amendment. For the MAD study, 3 mL of EDTA-K_2_ anticoagulated whole blood samples were collected at pre-dose and 0.25 h, 0.5 h, 0.75 h, 1 h, 1.5 h, 2 h, 4 h, 6 h, 8 h, 12 h, and 24 h post-dose for Day 1 post-dose, in addition to 48 h, 72 h, 96 h, and 120 h for Day 9 post-dose. In addition, whole blood samples at 0 h (pre-dose) from Days 7 and 8 were collected to determine the steady state. The collected blood samples were separated after centrifugation at 4 C, 1700 g for 10 min. The plasma was divided into two tubes and stored frozen at −80 C until analysis. HEC95468 in plasma was determined using a validated LC-MS/MS method. HEC95468-D5 was chosen as the internal standard. The calibration curves were linear over the concentration ranges from 0.2 to 2000 ng/mL. The maximum within-day precision was 7.25%, and the maximum between-day precision was 6.19%. Urine samples were collected at pre-dose (within 2 h) and 0–4 h, 4–8 h, 8–12 h, 12–24 h, 24–48 h, 48–72 h, 72–96 h, and 96–120 h post-dose in Group A of the 10 mg group (fasting state). Feces samples were collected at 0–120 h post-dose in the first treatment period of Group A of the 10 mg group. HEC95468 in urine and feces was determined using a validated LC-MS/MS method.

Major pharmacokinetic parameters, including area under the concentration–time curve (AUC), AUC from time zero (pre-dose) to 24 h post-dose (AUC_0–24h_), AUC from time zero (pre-dose) to the time of the last measurable concentration (AUC_0-t_), AUC from time zero (pre-dose) to infinity (AUC_0-∞_), terminal elimination half-life (t_1/2_), elimination rate constant (K_el_), apparent distribution volume (V_z_/F), apparent clearance rate (CL/F), and mean retention time (MRT), were calculated using a non-compartmental model by WinNonlin Software version 8.3 (Pharsight, Cary, NC, USA). The maximum observed plasma concentration (C_max_) and the time to maximum plasma concentration (T_max_) were based on the actual measured values. Metabolite identification and drug concentration determination (only for the 10 mg dose group) were performed on collected blood samples, urine, and feces to assess the metabolic characteristics of the drug. The amount of drug excretion and the cumulative excretion percentage were calculated separately. In multiple ascending dose studies, the degree of fluctuation (DF) and accumulation ratio at steady state (R) were also calculated.

### Pharmacodynamic assessment

Plasma vasoactive hormones (renin, angiotensin II, aldosterone, and noradrenaline) and cGMP were determined using a validated LC-MS/MS method. In addition, 6 mL of EDTA-K_2_ anticoagulated whole blood samples were collected at pre-dose and 1 h, 2 h, 4 h, 6 h, 8 h, and 24 h post-dose in the 0.5 mg, 1.25 mg, 2.5 mg, and 5 mg dose groups of the SAD study. The same time points were adopted in the pharmacodynamics study of the FE study. 12 h post-dose was added in the 15 mg, 20 mg, and 25 mg dose groups of the SAD study. The same time points were adopted in the MAD study on Days 1 and 9. The blood samples collected were separated after centrifugation at 4°C, 1700 g for 10 min. The plasma was divided into two centrifuge tubes (at least 0.5 mL each) and stored frozen at −80°C until analysis. Blood pressure (DBP and SBP) and heart rate were measured at the same time points as those of the plasma vasoactive hormones.

### Statistical analysis

For the safety assessments, the summary of TEAEs was provided with the number of cases, incidence, and number of events. Pharmacokinetic analysis was performed using WinNonlin Software version 8.3 (Pharsight, Cary, NC, USA). Descriptive statistics were expressed as the geometric mean and coefficient of covariance (CV%). In the SAD and MAD studies, the dose linear relationship was assessed using the power model. The regression equation was expressed as ln(PK) = α +β1×ln(Dose) after the logarithmic transformation of pharmacokinetic parameters and doses. When the 90% confidence interval of β for C_max_, AUC_0-t,_ and AUC_0-∞_ was within the acceptance interval of 0.8–1.25, C_max_, AUC_0-t_, or AUC_0-∞_ were dose-proportional. For the FE study, if the 90% confidence interval (90% CI) of the geometric mean ratio of the main pharmacokinetic parameters fell into the range of 0.8–1.25, it can be considered that food did not affect pharmacokinetics. For pharmacodynamic analysis, descriptive statistics were used to summarize the observed values at each time point after administration and their changes from the baseline, and a column chart of the mean and standard deviation over time was plotted. An analysis of variance was used to compare data across the groups.

## Results

### Demographic profile

A total of 134 eligible participants were enrolled and randomized in the study, including 67 in the SAD study, 18 in the FE and drug metabolism study, and 49 in the MAD study, respectively (Figure 1B). In the SAD study, two participants in the 0.5 mg group withdrew due to needle sickness before administration; three participants withdrew due to abnormal vital signs before administration (one in the 15 mg group and two in the 25 mg group). One in the 2.5 mg group withdrew on the 3rd day after administration due to personal reasons. Overall, 61 participants finished the SAD study. Eighteen participants finished the FE and drug metabolism study. Except for one participant who withdrew from the study due to abnormal vital signs before the first dose, 48 participants finished the MAD study. The baseline demographic characteristics of the participants at enrollment were similar among the treatment groups in terms of age, sex, ethnicity, and body mass index (BMI) (Table 1).

**TABLE 1:** Baseline demographic characteristics.

	SAD study								MAD study					FE study		
Dose, mg	0.5	1.25	2.5	5	15	20	25	P	1.25	2.5	5	7.5	P	10 (A)	10 (B)	P
n	6	6	8	8	8	6	6	14	10	10	10	10	8	8	8	2
Age, y																
Mean	28.8	34.2	28.1	30.1	26.6	27.7	36.8	31.1	27.4	27.4	30.2	30.7	31.8	31.3	30.3	24.0
SD	5.5	4.8	6.2	5.7	5.4	2.9	4.0	5.4	4.8	6.9	6.3	5.1	8.6	4.4	8.0	2.8
Sex, %																
Male	100	100	87.5	87.5	87.5	100	100	92.9	100	100	100	80	100	87.5	87.5	100
Female	0	0	12.5	12.5	12.5	0	0	7.1	0	0	0	20	0	12.5	12.5	0
Ethnicity, %																
Han	100	100	87.5	87.5	87.5	100	83.3	85.7	100	70	100	70	100	87.5	100	100
Others	0	0	12.5	12.5	12.5	0	16.7	14.3	0	30	0	30	0	12.5	0	0
BMI, kg/m^2^																
Mean	22.0	22.9	24.2	23.4	21.7	22.0	22.1	24.7	23.3	22.8	22.8	25.3	22.9	23.2	22.7	22.7
SD	1.5	3.3	2.7	2.9	3.3	3.0	2.2	2.0	3.1	2.9	2.9	2.1	2.8	3.3	2.6	2.4

SAD, single ascending dose; MAD, multiple ascending dose; FE, food effect; P, placebo; BMI, body mass index; SD, standard deviation.

### Safety and tolerability

In the SAD study (including 10 mg of fasting state), TEAEs occurred in every dose group. The incidence of drug-related TEAEs for 0.5 mg, 1.25 mg, 2.5 mg, 5 mg, 10 mg (fasting state), 15 mg, 20 mg, 25 mg, and placebo groups was 1/6 (16.7%), 2/6 (33.3%), 1/8 (12.5%), 7/8 (87.5%), 12/16 (75.0%), 7/8 (87.5%), 6/6 (100%), 5/6 (83.3%), and 6/16 (37.5%), respectively. The most common drug-related TEAEs (≥5% of all TEAEs) associated with HEC95468 were dizziness (16/64, 25.0%), headache (16/64, 25.0%), increased heart rate (16/64, 25.0%), decreased blood pressure (9/64, 14.1%), nasal congestion (6/64, 9.4%), nausea (5/64, 7.8%), and vomiting (4/64, 6.3%). Three cases of TEAEs were grade 2 TEAEs related to the drug, including two cases of nausea, one in the fasting state of the 10 mg group and the other in the 15 mg group, and one case of orthostatic hypotension in the fasting stage of the 10 mg group. All drug-related TEAEs were self-cured without treatment.

In the FE study, the incidence of drug-related TEAEs in the fasting 10 mg, fed 10 mg, and placebo groups was 12/16 (75.0%), 9/16 (56.3%), and 1/2 (50.0%), respectively. Similar to the SAD study, the most common drug-related TEAEs were dizziness, headache, increased heart rate, and decreased blood pressure. Two cases of grade 2 nausea occurred, one in the fasting state and one in the fed state; one case of grade 2 orthostatic hypotension occurred in the fasting state of the 10 mg group. All cases of grade 2 TEAEs were self-cured without treatment. Since two cases of grade 2 TEAEs occurred in the 10 mg group in the SAD study, the dose levels of 1.25 mg, 2.5 mg, 5 mg, and 7.5 mg were chosen as the doses in the MAD study. In addition, the clinically effective dose of riociguat in PAH indications is 3.0 mg/day–7.5 mg/day (Riociguat label, 2013); therefore, 7.5 mg was chosen as the highest dose level in the MAD study of HEC95468.

In the MAD study, a total of 32 (32/48, 66.7%) participants experienced drug-related TEAEs, including 5/10 (50.0%), 6/10 (60.0%), 7/10 (70.0%), 9/10 (90.0%), and 5/8 (62.5%) in the HEC95468 tablet 1.25 mg, 2.5 mg, 5 mg, 7.5 mg, and placebo groups, respectively. The most common drug-related TEAEs (≥10% of all TEAEs) associated with HEC95468 were foreign body sensation (17.5%, 7/40), decreased blood pressure (6/40, 15.0%), nausea (6/40, 15.0%), abdominal distension (6/40, 15.0%), headache (6/40, 15.0%), dizziness (5/40, 12.5%), gastroesophageal reflux disease (5/40, 12.5%), nasal congestion (4/40, 10.0%), diarrhea (4/40, 10.0%), and increased heart rate (4/40, 10.0%). Three cases of CTCAE grade 2 drug-related TEAEs occurred: one case of nausea in the 2.5 mg group; one case of foreign body sensation in the 7.5 mg group; and one case of abdominal distension in the 7.5 mg group. None of the grade 2 TEAEs required medical interventions.

All drug-related TEAEs were grade 1 or 2 adverse events. No adverse events led to study discontinuation. No serious adverse events were observed.

### Pharmacokinetic analysis

**FIGURE 2:**
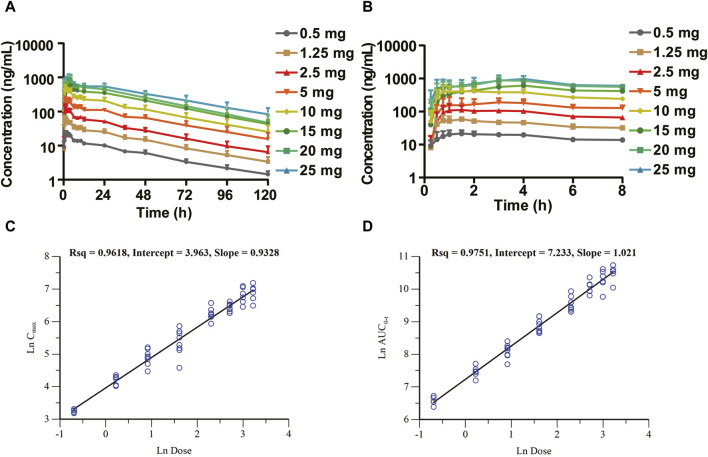
Mean plasma drug concentration–time (C–T) curves of the SAD study (semi-log) and dose-proportional analysis in the SAD study. **(A)** C–T curves at 0–120 h; **(B)** C–T curves at 0–8 h segment; **(C)** dose-proportional analysis of C_max_ vs. dose; and **(D)** dose-proportional analysis of AUC_0-t_ vs. dose.

The mean plasma drug concentration–time curves of the SAD study are shown in Figures 2A,B. The major pharmacokinetic parameters of HEC95468 in healthy volunteers after a single administration are summarized in Table 2. In the 10 mg group, only eight participants in the first cycle of the fasting state were included in the pharmacokinetic analysis. The median of time to maximum plasma concentration (T_max_) was 0.875–4.00 h in the dose range. The geometric mean of the maximum observed plasma concentration (C_max_) and the area under the concentration-time curve from time 0 to last (AUC_0-t_) were elevated with a dose increase from 0.5 mg to 25 mg. The parameters of mean retention time (MRT), time of half-life (t_1/2_), apparent distribution volume (V_z_/F), and clearance rate (CL/F) were similar in the eight dose groups. Dose proportionality was evaluated with the correlation coefficient, the regression slope, and confidence intervals. The correlation coefficient R^2^ was 0.9618 and 0.9751 for C_max_ vs dose and AUC_0-t_ vs dose, respectively (Figures 2C,D). The slope of C_max_ vs dose was 0.9328, and its 90% confidence interval was between 0.890 and 0.975. The slope of AUC_0-t_ vs. dose was 1.021, and its 90% confidence interval was (0.981, 1.05). Since the slope and the confidence interval were within the range of 0.8–1.25, C_max_ and AUC_0-t_ were dose-dependent in the dose range of 0.5–25 mg.

**TABLE 2:** Pharmacokinetic parameters of HEC95468 in the SAD study.

Parameter (Unit)	0.5 mg	1.25 mg	2.5 mg	5 mg	10 mg	15 mg	20 mg	25 mg
	n = 6	n = 6	n = 8	n = 8	n = 8^a^	n = 8	n = 6	n = 6
T_max_ (h)	1.25 (0.5, 3.0)	0.875 (0.75, 1.5)	1.25 (0.75, 4.0)	3.00 (1.0, 12.0)	1.13 (0.5, 4.0)	3.00 (1.5, 4.0)	3.00 (1.0, 4.0)	4.00 (0.5, 4.0)
C_max_ (ng/mL)	25.6 (5.08)	67.4 (14.2)	135 (24.4)	213 (42.6)	508 (18.4)	626 (12.1)	900 (25.4)	976 (25.1)
AUC_0–24h_ (h·ng/mL)	317 (9.35)	770 (15.0)	1590 (11.3)	2,949 (20.9)	6,051 (17.9)	9,512 (11.6)	12,922 (18.3)	14,187 (20.3)
AUC_0-t_ (h^.^ng/mL)	715 (11.8)	1746 (16.9)	3,507 (15.3)	7,268 (20.9)	13,606 (23.4)	23,668 (17.2)	29,469 (31.1)	37,096 (25.9)
AUC_0-∞_ (h^.^ng/mL)	789 (12.4)	1927 (17.1)	3,818 (18.8)	8,050 (24.5)	14,766 (26.7)	25,714 (17.5)	31,286 (34.7)	41,131 (29.9)
MRT (h)	48.3 (9.11)	48.1 (17.3)	44.5 (21.8)	49.7 (23.6)	45.3 (15.5)	46.7 (15.6)	40.5 (23.9)	50.0 (25.1)
t_1/2_ (h)	35.6 (13.1)	36.2 (17.4)	33.1 (21.6)	34.1 (22.2)	32.6 (18.1)	31.5 (21.5)	27.7 (22.9)	33.3 (23.0)
K_el_ (1/h)	0.0195 (13.1)	0.0191 (17.4)	0.0210 (21.6)	0.0203 (22.2)	0.0212 (18.1)	0.0220 (21.5)	0.0250 (22.9)	0.0208 (23.0)
V_Z_/F (L)	32.5 (19.0)	33.9 (23.9)	31.2 (12.2)	30.5 (19.3)	31.9 (15.9)	26.5 (30.1)	25.5 (16.6)	29.2 (19.5)
CL/F (L/h)	0.634 (12.4)	0.649 (17.1)	0.655 (18.8)	0.621 (24.5)	0.677 (26.7)	0.583 (17.5)	0.639 (34.7)	0.608 (29.9)

^a^ Since the pre-dose sample of the fasting treatment period of Group B was not below the quantification limit, only the result of the fasting treatment period of Group A (n = 8) was included in the pharmacokinetic analysis.

Data are expressed as the geometric mean (geometric %CV), except for T_max_, which is shown as median (min, max). Abbreviations: T_max_, time to maximum plasma concentration; C_max_, maximum observed plasma concentration; AUC_0–24h_, area under the concentration-time curve from time 0 to 24 h post-dose; AUC_0-t_, area under the concentration-time curve from time zero to the last measurable concentration; AUC_0-∞_, area under the concentration-time curve from time zero to infinity; MRT, mean retention time; t_1/2_, terminal elimination half-life; K_el_, elimination rate constant; Vz/F, apparent distribution volume; CL/F, clearance rate.

In the FE study, 10 mg of HEC95468 tablets were administered to 16 healthy volunteers under fasting and fed (high-fat and high-calorie meal) conditions. The geometric mean of C_max_ under fasting and fed conditions was 511 ng/mL and 373 ng/mL, respectively, and the geometric mean of AUC_0-t_ under fasting and fed conditions was 15,159 h·ng/mL and 14,325 h·ng/mL, respectively. Therefore, compared with fasting administration, the peak concentration of C_max_ in the fed state dropped by 27.41%, but AUC_0-t_ did not fluctuate under fasting and fed conditions. The median of T_max_ was 1.75 h and 3.00 h for fasting and fed state, respectively, and the median of T_max_ difference between the two groups was 0.875 h, with no statistical difference. The t_1/2_ was not significantly altered under the fed condition. The geometric mean of t_1/2_ under fasting and fed state was 33.9 h and 33.8 h, respectively.

**FIGURE 3:**
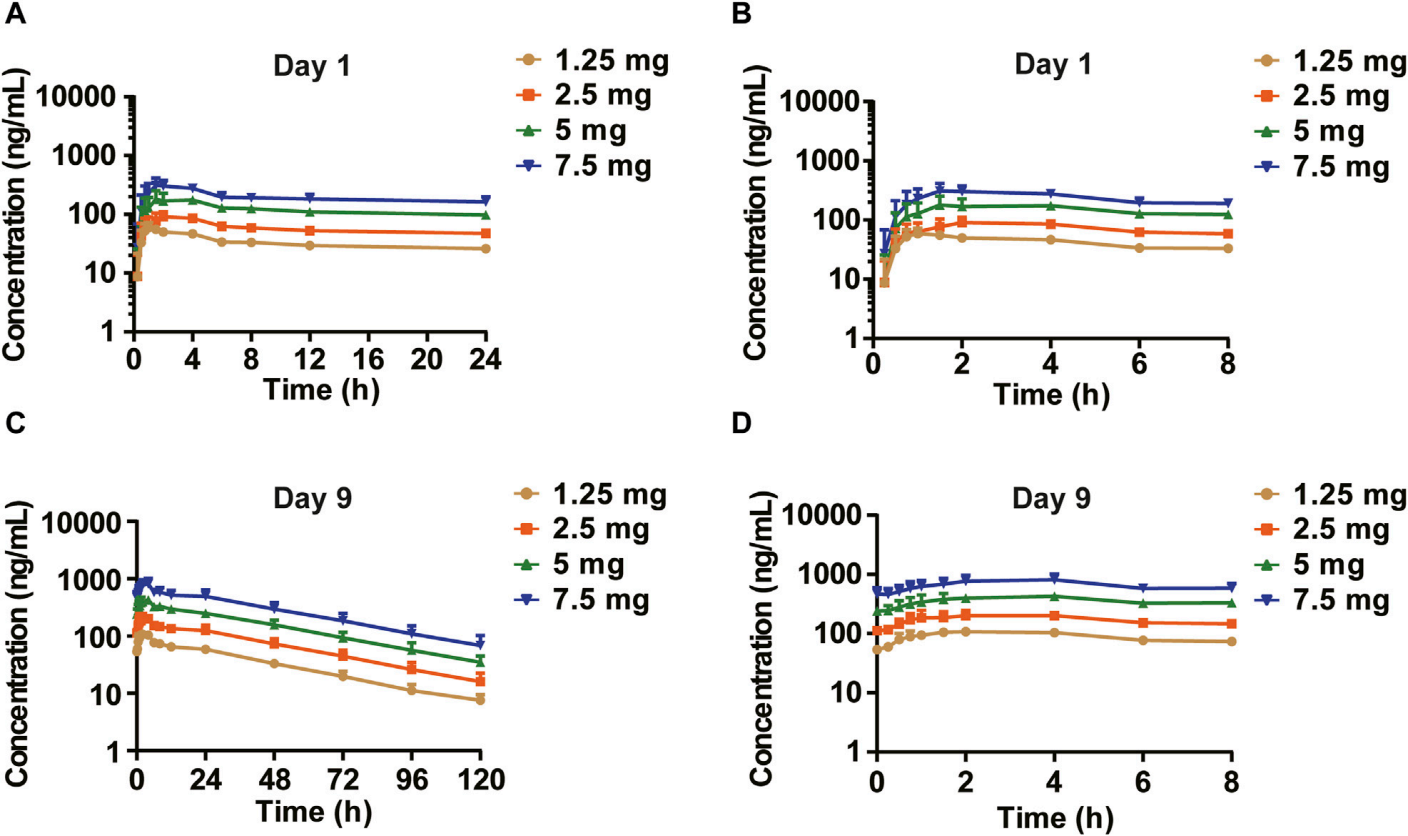
Mean plasma drug concentration–time curves of the MAD study (semi-log). **(A)** Day 1 (0–24 h); **(B)** Day 1 (0–8 h) segment; **(C)** Day 9 (0–120 h); and **(D)** Day 9 (0–8 h) segment.

**FIGURE 4:**
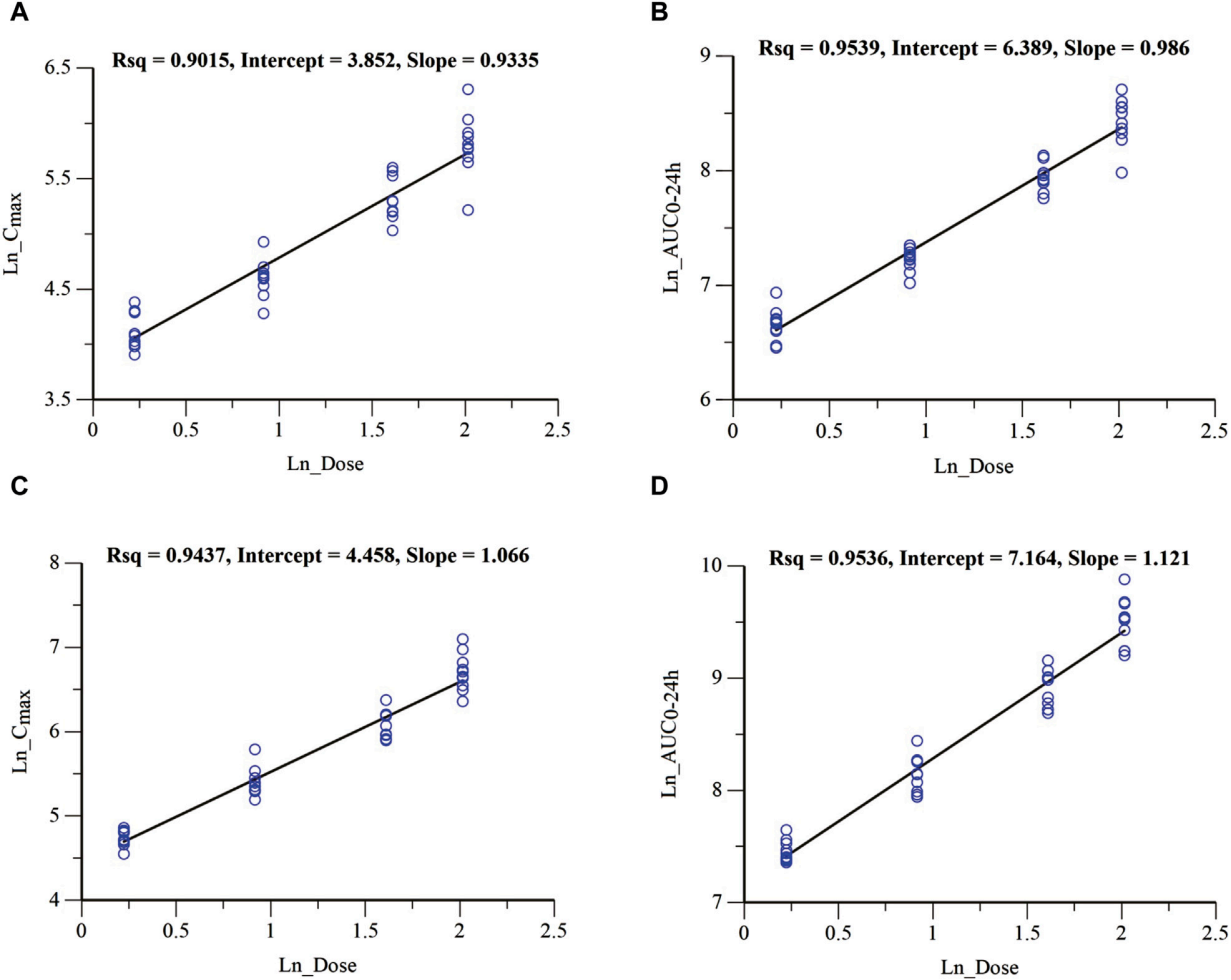
Dose-proportional analysis of C_max_ and AUC_0-24 h_ parameters in the MAD study. **(A)** C_max_ vs. dose of Day 1; **(B)** AUC_0-24 h_ vs. dose of Day 1; **(C)** C_max_ vs. dose of Day 9; and **(D)** AUC_0-24 h_ vs. dose of Day 9.

In the MAD study, the mean plasma drug concentration-time curves on Days 1 and 9 are shown in Figure 3. The pharmacokinetic parameters are shown in Table 3. C_max_, the minimum observed plasma concentration at steady state (C_ss_min_), and the area under the concentration–time curve from time 0–24 h (AUC_0–24h_) at steady state increased with the dose. C_max_ and AUC_0–24h_ of each dose group were accumulated after multiple administrations, and the accumulation factors ranged from 1.84–2.46 and 2.22–2.91, respectively. The geometric mean of the t_1/2_ was 31.6 h∼33.3 h. Compared with a single administration, there were no significant differences in the apparent volume of distribution V_z_/F, apparent clearance CL/F, and half-life t_1/2_ after multiple administrations in each dose group, suggesting that there was no significant change in the elimination characteristics of HEC95468 after multiple doses. Confidence interval criteria were also used to assess the dose linear relationship in the MAD study. On Day 1, the slope of C_max_ vs. dose and AUC_0–24h_ vs. dose was 0.9335 and 0.986, respectively (Figures 4A,B). On Day 9, the slope of C_max_ vs. dose and AUC_0–24h_ vs. dose was 1.066 and 1.121, respectively (Figures 4C,D). The correlation coefficient R^2^ of C_max_ and AUC_0–24h_ was 0.9015 and 0.9539 on Day 1 and 0.9437 and 0.9536 on Day 9, respectively. Therefore, C_max_ and AUC_0–24h_ were approximately dose-dependent in the dose range of 0.5–7.5 mg in the MAD study.

**TABLE 3:** Pharmacokinetic parameters of HEC95468 in the MAD study.

Parameter (Unit)	1.25 mg (n = 10)		2.5 mg (n = 10)		5 mg (n = 10)		7.5 mg (n = 10)	
	Day 1	Day 9	Day 1	Day 9	Day 1	Day 9	Day 1	Day 9
T_max_ (h)	1.00 (0.750,2.00)	2.00 (0.750,4.00)	2.00 (0.500,4.00)	2.00 (0.750,4.00)	1.50 (0.750,4.00)	4.00 (1.00,4.00)	1.25 (1.00,4.00)	4.00 (0.750,4.00)
C_max_ (ng/mL)	62.6 (16.7)	115 (9.56)	99.0 (16.8)	220 (16.9)	204 (19.0)	432 (16.6)	332 (28.6)	815 (22.3)
AUC_0–24h_ (h·ng/mL)	780 (13.9)	1729 (9.52)	1371 (9.89)	3,459 (16.2)	2,820 (11.8)	7,302 (16.9)	4,570 (20.9)	13,277 (22.2)
AUC_0-t_ (h^.^ng/mL)	775 (13.8)	4,025 (14.0)	1363 (9.85)	8,455 (21.0)	2,802 (11.8)	17,840 (20.0)	4,540 (20.8)	33,223 (28.0)
AUC_0-∞_ (h^.^ng/mL)	-	4,388 (14.9)	-	9,167 (23.1)	-	19,479 (21.4)	-	36,227 (30.2)
MRT (h)	-	47.5 (10.9)	-	50.5 (10.7)	-	50.9 (9.52)	-	52.4 (12.6)
t_1/2_ (h)	-	33.3 (12.7)	-	31.6 (14.8)	-	33.2 (11.1)	-	32.1 (13.4)
C_ss__min (ng/mL)	-	53.0 (10.3)	-	108 (19.5)	-	230 (19.7)	-	429 (27.0)
R_C_max_	-	1.84 (17.8)	-	2.22 (18.2)	-	2.12 (20.0)	-	2.46 (31.9)
R_AUC_0–24h_	-	2.22 (7.79)	-	2.52 (13.9)	-	2.59 (15.6)	-	2.91 (15.9)
DF (%)	-	85.2 (20.9)	-	76.4 (21.8)	-	66.3 (9.73)	-	68.4 (24.1)

Data are expressed as the geometric mean (geometric %CV), except for T_max_, which is shown as median (min, max). Abbreviations: T_max_, time to maximum plasma concentration; C_max_, maximum observed plasma concentration; AUC_0–24h_, area under the concentration-time curve from time 0 to 24 h post-dose; AUC_0-∞_, area under the concentration-time curve from time 0 to infinity; MRT, mean retention time; t_1/2_, terminal elimination half-life; C_ss__min, minimum observed plasma concentration at steady state; R_C_max_, accumulation ratio of C_max_; R_ AUC_0–24h_, accumulation ratio of AUC_0–24h_; DF, degree of fluctuation.

After oral administration of 10 mg of HEC95468 tablets, the cumulative total excretion rate in urine and feces was 14.95%, of which the average cumulative excretion rate of urine HEC95468 was 2.25% and the average cumulative excretion rate of fecal HEC95468 was 12.70%. In the results of metabolite identification, parent drugs accounted for 21.74% and 55.20% of the urine and fecal samples. The percentages of glucuronic acid conjugate in urine and fecal samples were 65.81% and 37.39%, respectively. Therefore, HEC95468 is eliminated in the form of the parent drug and glucuronic acid conjugates via urine and fecal excretion. For accurate results of the excretion route and Met ID, a clinical mass balance study with ^14^C-labeled HEC95468 will be conducted in the future.

### Pharmacodynamical analysis

**FIGURE 5:**
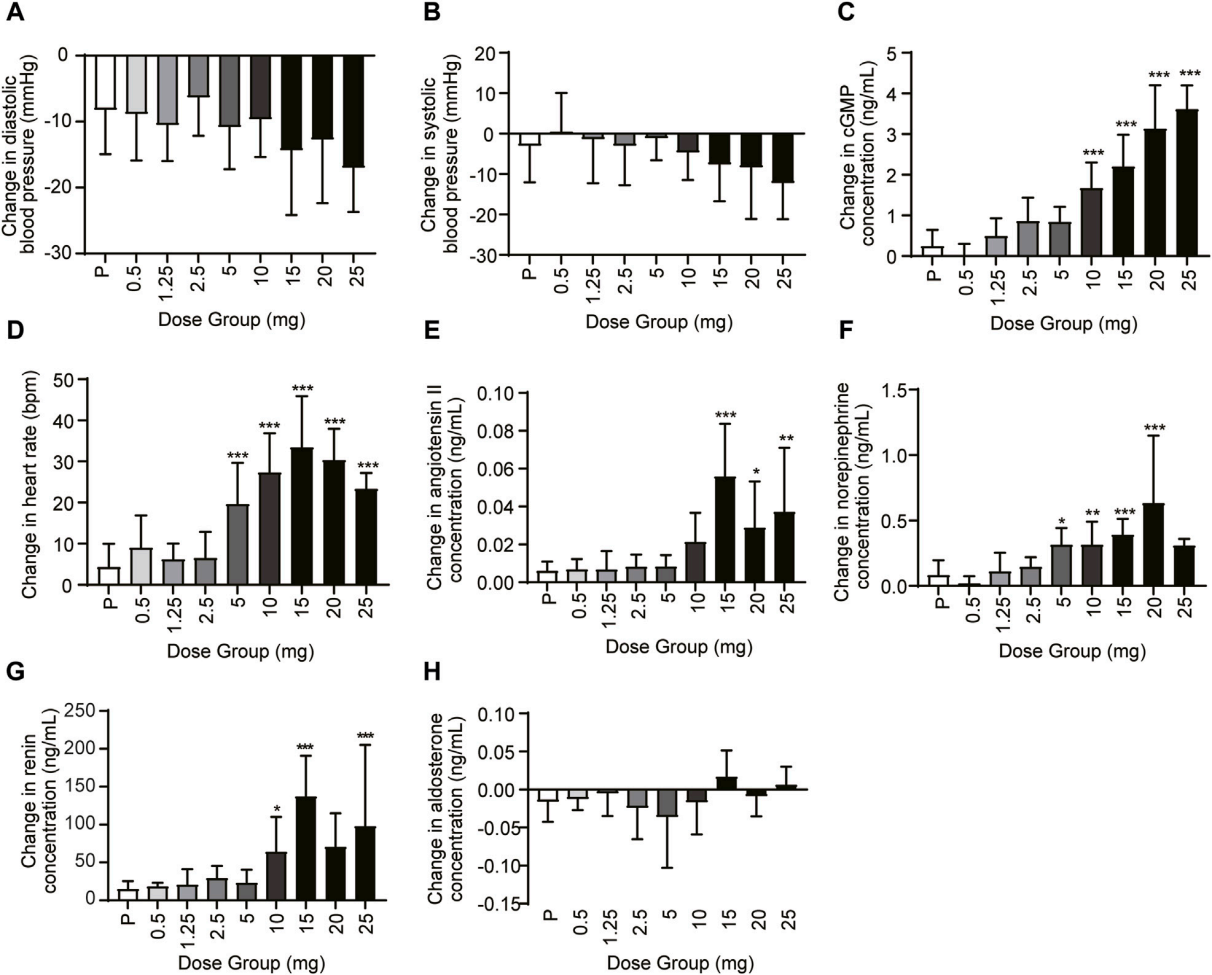
Representative pharmacodynamic profile of HEC95468 at 6 h post-dose in the SAD study. **(A)** Change in DBP from baseline. **(B)** Change in SBP from baseline. **(C)** Change in cGMP from baseline. **(D)** Change in the heart rate from baseline. **(E)** Change in angiotensin II from baseline. **(F)** Change in norepinephrine from baseline. **(G)** Change in renin concentration in the SAD study. **(H)** Change in aldosterone concentration in the SAD study; P, placebo. Data were presented as the mean ± SD. *p < 0.05 compared with placebo. **p < 0.01 compared with placebo. ***p < 0.001 compared with placebo.

In the SAD and MAD studies, blood pressure (DBP and SBP), heart rate, cGMP, and vasoactive hormones (angiotensin II, norepinephrine, renin, and aldosterone) were evaluated at different time points. Compared with the baseline, the DBP and the SBP decreased, especially in the 5–25 mg dose group 6–8 h post-dose (Figures 5A,B). The change in least squares mean of DBP and SBP from baseline was between −8.218 and −15.661 mmHg and between −3.667 and −11.041 mmHg, respectively. The mean cGMP concentration (standard deviation) was significantly induced, especially in the 10 mg, 15 mg, 20 mg, and 25 mg groups (*p* < 0.001) (Figure 5C). The least squares mean change in cGMP from baseline was −0.093–2.543 ng/mL in the 0.5–25 mg dose range. For the heart rate, HEC95468 induced a significant increase in the 5–25 mg of the SAD study (*p* < 0.001). The heart rate increased by 0.293–22.939 bpm in the 0.5–25 mg dose range (Figure 5D). Similar changes were observed in the plasma concentration of angiotensin II, norepinephrine (Figures 5E,F), and renin (Figure 5G) after a single administration of HEC95468. In addition, the plasma aldosterone level was not altered by HEC95468 (Figure 5H).

**FIGURE 6:**
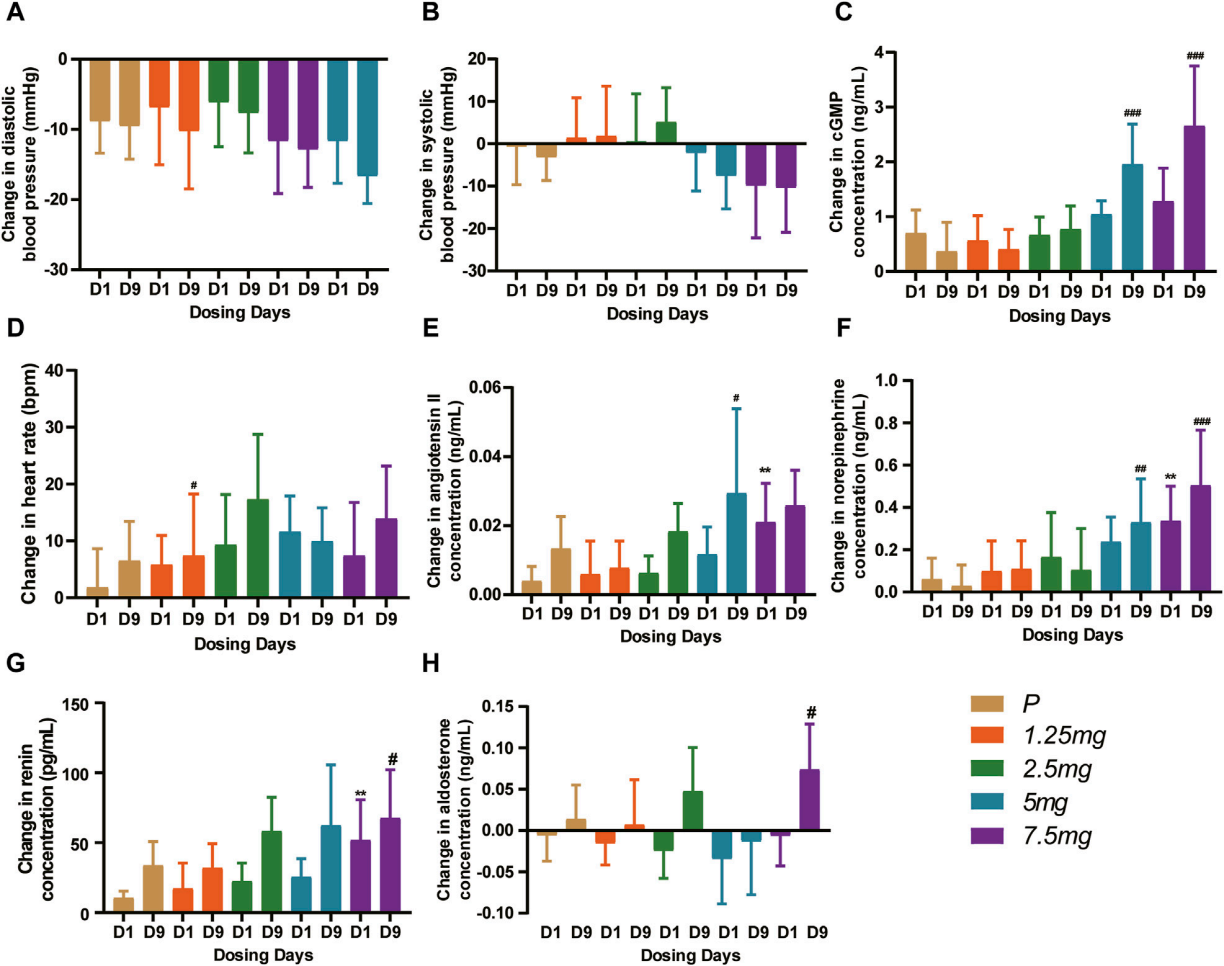
Representative pharmacodynamic profile of HEC95468 at 6 h post-dose in the MAD study. **(A)** Change in DBP from baseline. **(B)** Change in SBP from baseline. **(C)** Change in cGMP from baseline. **(D)** Change in the heart rate from baseline. **(E)** Change in angiotensin II from baseline. **(F)** Change in norepinephrine from baseline. **(G)** Change in renin concentration from baseline. **(H)** Change in aldosterone concentration from baseline. P, placebo. Data were presented as the mean ± SD. **p < 0.01 compared with placebo-Day 1. ^*#*^ *p* < 0.001 compared with placebo-Day 9. ^*##*^ *p* < 0.001 compared with placebo-Day 9. ^*###*^ *p* < 0.001 compared with placebo-Day 9.

In the MAD study, compared with the baseline, the DBP and the SBP decreased, especially in the 5 mg and 7.5 mg dose groups at 4–8 h post-dose (Figures 6A,B). After Day 1 and Day 9 administration, the least squares mean change in DBP from baseline was between −4.868 and −10.865 mmHg on Day 1 and between −5.636 and −11.577 mmHg on Day 9. The least squares mean change in SBP from baseline was between −0.429 and −9.089 mmHg on Day 1 and between 0.522 and −8.146 mmHg on Day 9. The mean cGMP concentration (standard deviation) was significantly induced, especially in the 5 mg and 7.5 mg groups on Day 9 (*p* < 0.001) (Figure 6C). The least squares mean change in cGMP from baseline after 9 consecutive days of administration was between 0.271 and 1.889 ng/mL. For the heart rate, HEC95468 induced a moderate increase in the MAD study. The heart rate increased by 3.805–9.994 bpm in the MAD study on Day 9 (Figure 6D). Similar changes were observed in the plasma concentration of angiotensin II, norepinephrine (Figures 6E,F), and renin (Figure 6G) after multiple administrations of HEC95468. In addition, plasma aldosterone levels were not altered by HEC95468 (Figure 6H). Since the induction of heart rate and vasoactive hormones was the compensatory effect of vasodilating agents, HEC95468 was an sGC stimulator with definite biological activity *in vivo*.

## Discussion and conclusion

In this study, we evaluated the safety and tolerability of an sGC stimulator, HEC95468, after single and multiple doses of administration and assessed the pharmacokinetics, pharmacodynamics, and food effect in healthy volunteers. Overall, HEC95468 was safe and tolerable; the *in vivo* exposure was dose-proportional over the dose range. The pharmacodynamic results were consistent with the biological mechanism of action of the drug. SBP and DBP decreased mildly. The heart rate, cGMP, and vasoactive hormones were elevated after single and multiple administrations of HEC95468 except aldosterone. These data supported further clinical trials of HEC95468 in the treatment of HF and PAH.

HEC95468 was well tolerated in the range of single administrations of 0.5 mg–25 mg and multiple administrations of 1.25 mg–7.5 mg. The adverse events were mostly mild and transient. A higher proportion of HEC95468-treated participants reported mild headaches, dizziness, decreased blood pressure, increased heart rate, and gastrointestinal (GI)-related TEAEs, similar to the sGC stimulators riociguat and vericiguat (Frey et al., 2008a; Zhao et al., 2016; Boettcher et al., 2021). The most common adverse reactions (≥3%) of riociguat were headache, dyspepsia/gastritis, dizziness, nausea, diarrhea, hypotension, vomiting, anemia, gastroesophageal reflux, and constipation. The most common adverse reactions of vericiguat reported in ≥5% were hypotension and anemia. The occurrence of the above adverse events correlated with the dose level and was related to the NO-sGC-cGMP signal pathway, suggesting that the above adverse events need to be monitored in subsequent clinical trials. Notably, neither increases in bleeding time nor any bleeding events were reported in the HEC95468-treated participants in this study, although stimulation of NO–sGC–cGMP signaling has been shown to inhibit platelet function *in vitro* and serious bleeding events were reported more with riociguat treatment in phase III clinical trials (Rosenkranz et al., 2015). Therefore, HEC95468 has a similar safety profile compared with riociguat and vericiguat. No unexpected TEAEs occurred in the study.

HEC95468 exhibited a favorable pharmacokinetic profile in healthy volunteers. Moderate apparent systemic clearance results in a medium end-stage half-life of approximately 1–2 days. The peak: trough ratio was approximately 2 after QD dosing for 9 consecutive days. The steady state of C_ss_ exposure restrained pharmacodynamic fluctuations over 24 h. HEC95468 also showed dose-proportional exposure (C_max_ and AUC_0-t_) with moderate individual differences in liver-driven clearance primarily via feces, suggesting predictable exposure and a lower likelihood of dose adjustment in patients with impaired kidneys. HEC95468 exhibited a long half-life of elimination *in vivo*, which was guaranteed once daily in clinical application. The pharmacokinetic profile of HEC95468 was better than that of riociguat and vericiguat (Frey et al., 2008b; Zhao et al., 2016; Hanrahan et al., 2019; van Kraaij et al., 2023).

**FIGURE 7:**
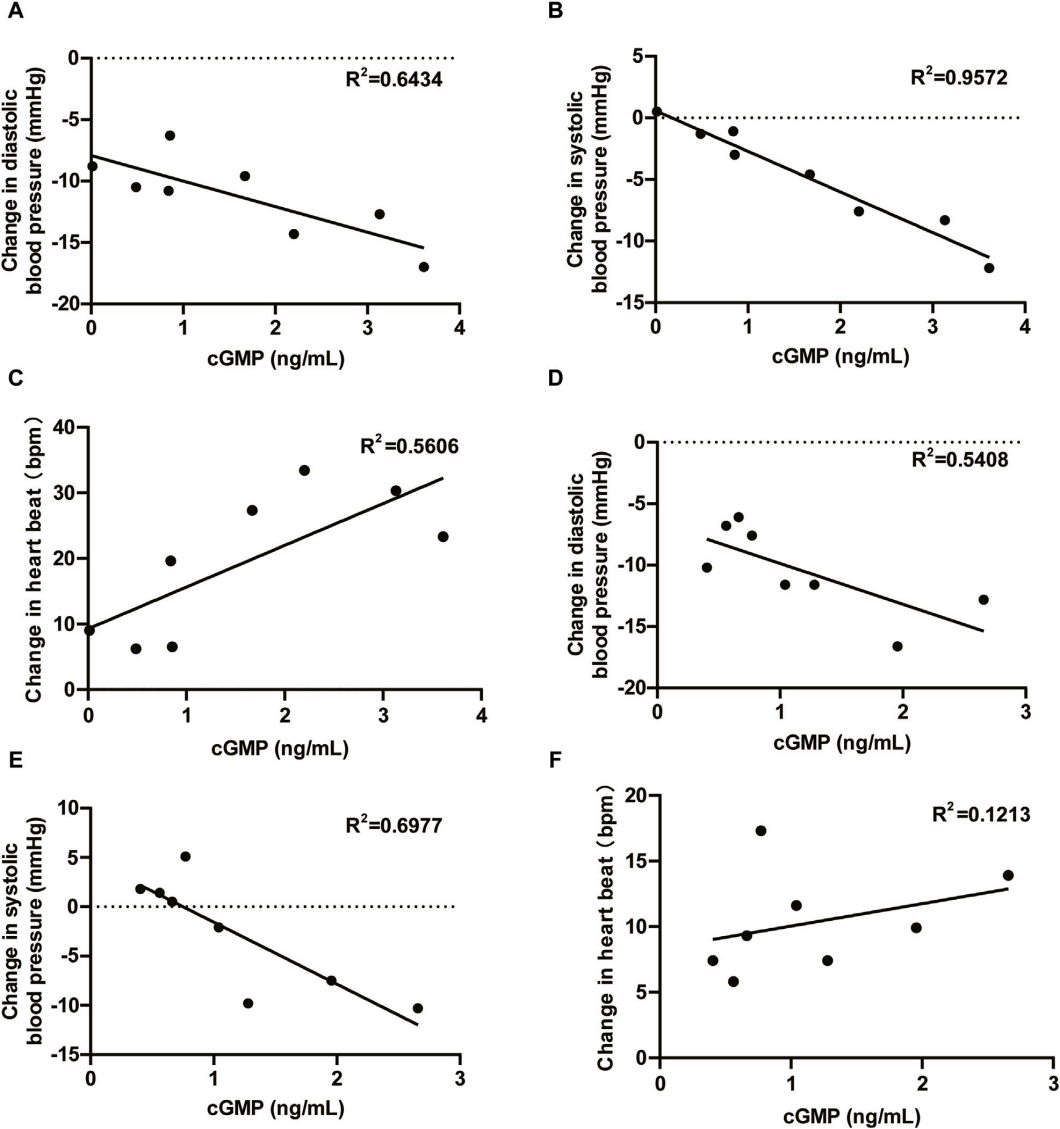
Correlations between cGMP and blood pressure and cGMP and heartbeat. **(A)** DBP vs. cGMP in the SAD study; **(B)** SBP vs. cGMP in the SAD study; **(C)** HR vs. cGMP in the SAD study; **(D)** DBP vs. cGMP in the MAD study; **(E)** SBP vs. cGMP in the MAD study; and **(F)** HR vs. cGMP in the MAD study. DBP, diastolic blood pressure; SBP, systolic blood pressure; HR, heartbeat; R^2^, coefficient of correlation.

The administration of HEC95468 led to a reduction in diastolic blood pressure and systolic blood pressure, which was consistent with other marketed sGC stimulators (Boettcher et al., 2021; Boettcher et al., 2022). The correlation between cGMP and BP or heart rate has been plotted and added in Figure 7. In the SAD study, the coefficient of correlation between cGMP and BP or heart rate was >0.5 in Figures 7A–C. In the MAD study, the coefficient of correlation between cGMP and BP was >0.5 in Figures 7D,E and the coefficient of correlation between cGMP and heartbeat was <0.5 in Figure 7F. Hemodynamic regulation in these healthy young participants resulted in the induction of plasma cGMP levels, heart rate, and vasoactive hormones (except noradrenaline). Although plasma cGMP levels were thought of as an overspill of intracellular cGMP with unknown biological activity, the dose-dependent increases from baseline were still informative. The increase in cGMP and vasoactive hormones indicated target engagement at the tissue level (Buglioni and Burnett, 2016). In addition, changes in heart rate were regarded as a sensitive parameter for indirect estimation of a vasodilating agent on the cardiovascular system in healthy young volunteers (Frey et al., 2008b). To compensate for the reduction in blood pressure, the cardiovascular system stimulates the heart rate, leading to an increased cardiac output to keep the blood pressure stable. The observed increase in heart rate was dose-dependent at single oral doses of HEC95468 of 5.0–20 mg, as expected. The induction of vasoactive hormones demonstrated the extent of compensational efforts (Emdin et al., 2020). A dose-dependent increase in renin levels was observed at doses of 10–25 mg, in parallel with an increase in angiotensin II or norepinephrine. Therefore, steady and mechanism-mediated pharmacodynamic effects were positively synchronized with the pharmacokinetic profile of HEC95468. In the SAD and MAD studies, a placebo effect was observed (Figure 5; Figure 6). White-coat hypertension or postprandial hypotension could be the reason for the placebo effect. When measuring the blood pressure at baseline, participants may encounter white-coat hypertension. For the following time points, participants may get used to the white coats of doctors and nurses. Therefore, the blood pressure decreased compared with that at baseline. In addition, postprandial hypotension may occur at 6 hours post-dose, which is 2 p.m. when participants just had lunch.

HF is a growing public health issue and has become a major cause of hospitalization, morbidity, and mortality (Ziaeian and Fonarow, 2016). It is the most costly cardiovascular disorder and the leading cause of hospitalization in patients older than 65 years of age (Savarese et al., 2023). In patients with HF, the myocardium is unable to maintain a cardiac output sufficient to meet the demands of peripheral circulation. If left untreated, the outcome is death (Wang et al., 2021). The decompensated heart is associated with a severe degree of endothelial dysfunction. In the healthy endothelium, vascular nitric oxide (NO) activates a key signal-transduction enzyme, sGC, resulting in the conversion of guanosine triphosphate (GTP) into the second messenger cGMP (Armstrong et al., 2018). This NO–sGC–cGMP pathway is responsible for vasodilation. In participants with heart failure, the pathway is disrupted by a decreased bioavailability of NO, resulting in insufficient vasodilation. Therefore, sGC stimulators activate sGC and provide considerable therapeutic advantages in HF patients (Rüdebusch et al., 2022). Thus, HEC95468 may benefit the treatment of HF via the induction of the NO-sGC-cGMP pathway.

In conclusion, HEC95468 was safe and tolerable in healthy volunteers with a favorable pharmacokinetic and pharmacodynamic profile. The data supported the use of HEC95468 as a candidate for the treatment of HF and PAH in further clinical trials.

## Data Availability

The original contributions presented in the study are included in the article/Supplementary Material; further inquiries can be directed to the corresponding author.

## References

[B1] Armstrong P. W. Roessig L. Patel M. J. Anstrom K. J. Butler J. Voors A. A. (2018). A multicenter, randomized, double-blind, placebo-controlled trial of the efficacy and safety of the oral soluble guanylate cyclase stimulator: the VICTORIA trial. JACC Hear. Fail. 6, 96–104. 10.1016/j.jchf.2017.08.013 29032136

[B2] Boettcher M. Düngen H. D. Donath F. Mikus G. Werner N. Thuermann P. A. (2022). Vericiguat in combination with short-acting nitroglycerin in patients with chronic coronary syndromes: the randomized, phase ib, VENICE study. Clin. Pharmacol. Ther. 111, 1239–1247. 10.1002/cpt.2574 35258101 PMC9310564

[B3] Boettcher M. Thomas D. Mueck W. Loewen S. Arens E. Yoshikawa K. (2021). Safety, pharmacodynamic, and pharmacokinetic characterization of vericiguat: results from six phase I studies in healthy subjects. Eur. J. Clin. Pharmacol. 77, 527–537. 10.1007/s00228-020-03023-7 33125516 PMC7935833

[B4] Buglioni A. Burnett J. C. (2016). New pharmacological strategies to increase cGMP. Annu. Rev. Med. 67, 229–243. 10.1146/annurev-med-052914-091923 26473417

[B5] Butler J. Usman M. S. Anstrom K. J. Blaustein R. O. Bonaca M. P. Ezekowitz J. A. (2022). Soluble guanylate cyclase stimulators in patients with heart failure with reduced ejection fraction across the risk spectrum. Eur. J. Heart Fail. 24, 2029–2036. 10.1002/ejhf.2720 36250238

[B6] Cai A. Zheng C. Qiu J. Fonarow G. C. Lip G. Y. H. Feng Y. (2023). Prevalence of heart failure stages in the general population and implications for heart failure prevention: reports from the China Hypertension Survey 2012–15. Eur. J. Prev. Cardiol. 30, 1391–1400. 10.1093/eurjpc/zwad223 37410587

[B7] Emdin M. Aimo A. Castiglione V. Vergaro G. Georgiopoulos G. Saccaro L. F. (2020). Targeting cyclic guanosine monophosphate to treat heart failure: JACC review topic of the week. J. Am. Coll. Cardiol. 76, 1795–1807. 10.1016/j.jacc.2020.08.031 33032741

[B8] Frey R. Mück W. Unger S. Artmeier-Brandt U. Weimann G. Wensing G. (2008a). Single-dose pharmacokinetics, pharmacodynamics, tolerability, and safety of the soluble guanylate cyclase stimulator BAY 63-2521: an ascending-dose study in healthy male volunteers. J. Clin. Pharmacol. 48, 926–934. 10.1177/0091270008319793 18519919

[B9] Frey R. Mück W. Unger S. Artmeier-Brandt U. Weimann G. Wensing G. (2008b). Pharmacokinetics, pharmacodynamics, tolerability, and safety of the soluble guanylate cyclase activator cinaciguat (BAY 58-2667) in healthy male volunteers. J. Clin. Pharmacol. 48, 1400–1410. 10.1177/0091270008322906 18779378

[B10] Greene S. J. Bauersachs J. Brugts J. J. Ezekowitz J. A. Filippatos G. Gustafsson F. (2023a). Management of worsening heart failure with reduced ejection fraction: JACC focus seminar 3/3. J. Am. Coll. Cardiol. 82, 559–571. 10.1016/j.jacc.2023.04.057 37532426

[B11] Greene S. J. Bauersachs J. Brugts J. J. Ezekowitz J. A. Lam C. S. P. Lund L. H. (2023b). Worsening heart failure: nomenclature, epidemiology, and future directions: JACC review topic of the week. J. Am. Coll. Cardiol. 81, 413–424. 10.1016/j.jacc.2022.11.023 36697141

[B12] Hanrahan J. P. Wakefield J. D. Wilson P. J. Mihova M. Chickering J. G. Ruff D. (2019). A randomized, placebo-controlled, multiple-ascending-dose study to assess the safety, tolerability, pharmacokinetics, and pharmacodynamics of the soluble guanylate cyclase stimulator praliciguat in healthy subjects. Clin. Pharmacol. Drug Dev. 8, 564–575. 10.1002/cpdd.627 30422390

[B13] Inciardi R. M. Bonelli A. Biering-Sorensen T. Cameli M. Pagnesi M. Lombardi C. M. (2022). Left atrial disease and left atrial reverse remodelling across different stages of heart failure development and progression: a new target for prevention and treatment. Eur. J. Heart Fail. 24, 959–975. 10.1002/ejhf.2562 35598167 PMC9542359

[B15] Lombardi C. M. Cimino G. Pagnesi M. Dell’Aquila A. Tomasoni D. Ravera A. (2021). Vericiguat for heart failure with reduced ejection fraction. Curr. Cardiol. Rep. 23, 144–147. 10.1007/s11886-021-01580-6 34410527 PMC8376697

[B16] Nieminen M. S. Böhm M. Cowie M. R. Drexler H. Filippatos G. S. Jondeau G. (2005). Executive summary of the guidelines on the diagnosis and treatment of acute heart failure: the task force on acute heart failure of the European society of cardiology. Eur. Heart J. 26, 384–416. 10.1093/eurheartj/ehi044 15681577

[B17] Olivotto I. Udelson J. E. Pieroni M. Rapezzi C. (2023). Genetic causes of heart failure with preserved ejection fraction: emerging pharmacological treatments. Eur. Heart J. 44, 656–667. 10.1093/eurheartj/ehac764 36582184

[B18] Packer M. Butler J. (2023). Similarities and distinctions between acetazolamide and sodium–glucose cotransporter 2 inhibitors in patients with acute heart failure: key insights into ADVOR and EMPULSE. Eur. J. Heart Fail. 1–7, 1537–1543. 10.1002/ejhf.2968 37403655

[B19] Packer M. Wilcox C. S. Testani J. M. (2023). Critical analysis of the effects of SGLT2 inhibitors on renal tubular sodium, water and chloride homeostasis and their role in influencing heart failure outcomes. Circulation 148, 354–372. 10.1161/CIRCULATIONAHA.123.064346 37486998 PMC10358443

[B14] Riociguat label (2013). Riociguat label. Silver Spring, MD: US FDA. Available at: www.accessdata.fda.gov/scripts/cder/daf/index.cfm?event=overview.process&ApplNo=204819 .

[B20] Rosenkranz S. Ghofrani H. A. Beghetti M. Ivy D. Frey R. Fritsch A. (2015). Riociguat for pulmonary arterial hypertension associated with congenital heart disease. Heart 101, 1792–1799. 10.1136/heartjnl-2015-307832 26135803 PMC4680166

[B21] Rüdebusch J. Benkner A. Nath N. Fleuch L. Kaderali L. Grube K. (2022). Stimulation of soluble guanylyl cyclase (sGC) by riociguat attenuates heart failure and pathological cardiac remodelling. Br. J. Pharmacol. 179, 2430–2442. 10.1111/bph.15333 33247945

[B22] Sachdev V. Sharma K. Keteyian S. J. Alcain C. F. Desvigne-Nickens P. Fleg J. L. (2023). Supervised exercise training for chronic heart failure with preserved ejection fraction: a scientific statement from the American heart association and American college of cardiology. Circulation 147, E699–E715. 10.1161/CIR.0000000000001122 36943925 PMC12019885

[B23] Savarese G. Becher P. M. Lund L. H. Seferovic P. Rosano G. M. C. Coats A. J. S. (2023). Global burden of heart failure: a comprehensive and updated review of epidemiology. Cardiovasc. Res. 118, 3272–3287. 10.1093/cvr/cvac013 35150240

[B24] Tsao C. W. Aday A. W. Almarzooq Z. I. Anderson C. A. M. Arora P. Avery C. L. (2023). Heart disease and stroke statistics—2023 update: a report from the American heart association. Circulation 147, e93–e621. 10.1161/CIR.0000000000001123 36695182 PMC12135016

[B25] van Kraaij S. J. W. Gal P. Borghans L. G. J. M. Klaassen E. S. Dijkstra F. Winrow C. (2023). First-in-human trial to assess safety, tolerability, pharmacokinetics, and pharmacodynamics of zagociguat (CY6463), a CNS-penetrant soluble guanylyl cyclase stimulator. Clin. Transl. Sci. 16, 1381–1395. 10.1111/cts.13537 37118895 PMC10432884

[B26] Varughese S. (2007). Management of acute decompensated heart failure. Crit. Care Nurs. Q. 30, 94–103. 10.1097/01.CNQ.0000264253.52381.2a 17356351

[B27] Wang H. Chai K. Du M. Wang S. Cai J. P. Li Y. (2021). Prevalence and incidence of heart failure among urban patients in China: a national population-based analysis. Circ. Hear. Fail. 14, E008406. 10.1161/CIRCHEARTFAILURE.121.008406 34455858

[B28] Yogasundaram H. Zheng Y. Ly E. Ezekowitz J. Ponikowski P. Lam C. S. P. (2023). Relationship between baseline electrocardiographic measurements and outcomes in patients with high-risk heart failure: insights from the VerICiguaT Global Study in Subjects with Heart Failure with Reduced Ejection Fraction (VICTORIA) trial. Eur. J. Heart Fail 25, 1822–1830. 10.1002/ejhf.3021 37655679

[B29] Zhao X. Wang Z. Wang Y. Zhang H. Blode H. Yoshikawa K. (2016). Pharmacokinetics of the soluble guanylate cyclase stimulator riociguat in healthy young Chinese male non-smokers and smokers: results of a randomized, double-blind, placebo-controlled study. Clin. Pharmacokinet. 55, 615–624. 10.1007/s40262-015-0337-4 26507720

[B30] Ziaeian B. Fonarow G. C. (2016). Epidemiology and aetiology of heart failure. Nat. Rev. Cardiol. 13, 368–378. 10.1038/nrcardio.2016.25 26935038 PMC4868779

